# Gastrotomy

**Published:** 1860-05

**Authors:** 


*Gastrotomy.*—Some of our readers may remember that about five years ago Dr. John Bell, of Wapello, Iowa, reported, in the *Iowa Medical and Surgical Journal*, the extraction of a bar of lead from the stomach—the case being operated upon by himself, and resulting in recovery. The report of the case is produced in the *Boston Medical and Surgical Journal* for January 19th. The bar of lead was 10¾ inches long, and weighed 9¼ ounces. The incision into the stomach was made on the left anterior side, and about parallel with the pylorus. The patient made a good recovery, and was discharged on the 15th day after the operation.—*American Medical Monthly.*

